# Working, declarative and procedural memory in specific language impairment

**DOI:** 10.1016/j.cortex.2011.06.001

**Published:** 2012-10

**Authors:** Jarrad A.G. Lum, Gina Conti-Ramsden, Debra Page, Michael T. Ullman

**Affiliations:** a Deakin University, Melbourne, Australia; b The University of Southern Denmark, Odense, Denmark; c The University of Manchester, Manchester, UK; d Georgetown University, Washington D.C., USA

**Keywords:** Specific language impairment (SLI), Working memory, Procedural memory, Declarative memory, Procedural Deficit Hypothesis (PDH)

## Abstract

According to the Procedural Deficit Hypothesis (PDH), abnormalities of brain structures underlying procedural memory largely explain the language deficits in children with specific language impairment (SLI). These abnormalities are posited to result in core deficits of procedural memory, which in turn explain the grammar problems in the disorder. The abnormalities are also likely to lead to problems with other, non-procedural functions, such as working memory, that rely at least partly on the affected brain structures. In contrast, declarative memory is expected to remain largely intact, and should play an important compensatory role for grammar. These claims were tested by examining measures of working, declarative and procedural memory in 51 children with SLI and 51 matched typically-developing (TD) children (mean age 10). Working memory was assessed with the Working Memory Test Battery for Children, declarative memory with the Children’s Memory Scale, and procedural memory with a visuo-spatial Serial Reaction Time task. As compared to the TD children, the children with SLI were impaired at procedural memory, even when holding working memory constant. In contrast, they were spared at declarative memory for visual information, and at declarative memory in the verbal domain after controlling for working memory and language. Visuo-spatial short-term memory was intact, whereas verbal working memory was impaired, even when language deficits were held constant. Correlation analyses showed neither visuo-spatial nor verbal working memory was associated with either lexical or grammatical abilities in either the SLI or TD children. Declarative memory correlated with lexical abilities in both groups of children. Finally, grammatical abilities were associated with procedural memory in the TD children, but with declarative memory in the children with SLI. These findings replicate and extend previous studies of working, declarative and procedural memory in SLI. Overall, we suggest that the evidence largely supports the predictions of the PDH.

## 1 Introduction

Children with specific language impairment (SLI) have below-average language abilities despite normal intellectual and sensory functioning (American Psychiatric Association, 2000; World Health Organization, 2004). A number of proposals have suggested that the language problems in SLI are related to memory deficits in the disorder (for recent reviews, see Montgomery et al., 2010; Ullman and Pierpont, 2005). Most theoretical and empirical work examining the relation between memory and language in SLI has focused on working memory (e.g., Archibald and Gathercole, 2006a, 2007; Ellis Weismer et al., 1999; Marton and Schwartz, 2003). However, it has also been proposed that the language problems in SLI may be largely explained by procedural memory (Ullman, 2004; Ullman and Pierpont, 2005). According to the Procedural Deficit Hypothesis (PDH), SLI is associated with abnormalities of brain structures underlying procedural memory, in particular portions of frontal/basal-ganglia circuits (Ullman and Pierpont, 2005). Other functions that rely on portions of these brain structures, including working memory, are also likely to be impaired. In contrast, declarative memory is posited to remain largely intact. The present study examined these predictions by testing for (1) group differences between SLI and typically-developing (TD) children in multiple measures of working, declarative, and procedural memory; and (2) associations between these memory measures and both lexical and grammatical abilities within the same set of SLI and TD children.

### 1.1 The working, declarative and procedural memory systems and their interactions

Considerable research suggests the existence of at least partly distinct memory systems in the brain, including working, declarative and procedural memory (Baddeley, 2003; Packard, 2009; Squire, 2004). Working memory supports the short-term storage and processing or manipulation of information. Agreement has yet to be reached concerning the cognitive architecture of this memory system. In Baddeley’s model, a “central executive” regulates the flow of information into two modality-specific slave systems: the phonological loop and visuo-spatial sketchpad, which temporarily store verbal and visuo-spatial information, respectively (Baddeley, 2000; Baddeley, 2002). According to Cowan (1988, 1995), the “focus of attention” holds a limited number of items, which are an activated subset of long-term memories.

Working memory is supported by multiple neural structures (D’Esposito, 2007). Prefrontal cortex, in particular dorsolateral prefrontal cortex (e.g., BA 46), plays an important role in the central executive and attentional processes posited by Baddeley and Cowan (Curtis and D’Esposito, 2003; Wager and Smith, 2003). The basal ganglia also seem to play a role in these executive/attentional working memory functions (McNab and Klingberg, 2007; O’Reilly and Frank, 2006). One proposal is that the connections from the basal ganglia to prefrontal cortex act as a gating system that allows information held in working memory to be updated with relevant information from long-term memory or from the environment (Frank et al., 2001; McNab and Klingberg, 2007). The storage of the information held in working memory seems to depend at least in part on Broca’s area and left posterior parietal cortex for verbal information, and right parietal and occipital cortex for visuo-spatial information (Gathercole, 1999; Smith and Jonides, 1998).

Whereas working memory maintains information in the order of seconds, declarative and procedural memory support long-term knowledge, and can store information for years. Declarative memory underlies the encoding, storage and retrieval of knowledge about personal experiences (episodic knowledge) and general knowledge about the world (semantic knowledge) (Eichenbaum, 2004; Squire, 2004). Evidence also suggests that it underlies lexical knowledge, including word forms and meanings (Ullman, 2001; Ullman, 2004). The system may be specialised for learning arbitrary pieces of information and binding them together. Information learned in this system is at least partly, though not completely, explicit (Chun, 2000; Daselaar et al., 2006). Learning by the declarative memory system can be achieved following a single exposure, though it is strengthened by multiple exposures.

Declarative memory is principally supported by the hippocampus and nearby structures in the medial temporal lobes (Eichenbaum, 2004; Squire et al., 2004). These structures underlie the learning and consolidation of new information, as well as the retrieval of this information. There appears to be some degree of hemispheric specialisation, with structures in the left medial temporal lobe more important for language-related material and those in the right hemisphere more important for visual and visuo-spatial information (Glosser et al., 1995; Jambaqué et al., 2007). Over the course of months to years, information eventually becomes largely independent of medial temporal lobe structures, and comes to rely instead primarily on neocortex. Different neocortical areas underlie different types of knowledge. For example, phonological word forms rely on posterior superior temporal cortex, whereas visual information depends on areas near visual cortices (Indefrey and Cutler, 2004; Martin and Chao, 2001). Other brain structures also play roles in declarative memory, including portions of prefrontal cortex (e.g., in the region of Brodmann’s Areas 45/47) in memory selection or retrieval (Buckner and Wheeler, 2001; Wagner et al., 1998). Note that we use the term “declarative memory system” to refer to the *entire* brain system involved in the learning and use of the relevant knowledge (Eichenbaum, 2000; Ullman, 2004), not just to those parts underlying learning and consolidation.

The procedural memory system is one of several brain systems involved in the implicit acquisition, storage and use of knowledge (Gabrieli, 1998; Squire and Zola, 1996; Willingham, 1998). This system underlies a variety of perceptual, motor and cognitive skills. For example, it subserves sequencing (Fletcher et al., 2005; Willingham et al., 2002), navigation (e.g., “response” learning and strategies in rodents) (Packard, 2009), and probabilistic categorisation (Knowlton et al., 1996; Poldrack et al., 2001). Evidence has been presented to suggest that procedural memory subserves the learning and use of rule-governed aspects of grammar, across syntax, morphology and phonology (Ullman, 2001, 2004; Ullman and Pierpont, 2005). Learning in procedural memory is slower than in declarative memory; it proceeds gradually, as stimuli are repeated and skills practiced. However, once this knowledge has been acquired, skills can be executed rapidly.

Although the neural bases of procedural memory are less well understood than those of declarative memory, evidence suggests that this system is supported by a network of brain structures that includes the basal ganglia, cerebellum and portions of frontal cortex, including premotor cortex and posterior parts of Broca’s area (e.g., BA 44) (Gabrieli, 1998; Knowlton et al., 1996; Robertson et al., 2001; Ullman, 2004; Ullman and Pierpont, 2005). The basal ganglia may play a particularly important role in learning and consolidation, while the frontal regions may be more important in the processing of already-learned procedures (Ullman, 2004, 2006b).

Though working, declarative and procedural memory systems are at least partly distinct, they also interact in various ways. Here we focus on two of these types of interactions. First, evidence suggests that working memory is closely related to declarative memory. For example, prefrontal structures that underlie the retrieval of information from declarative memory (the region of BA 45/47) also support working memory (Braver et al., 2001; Buckner et al., 1999; Simons and Spiers, 2003). And dorsolateral prefrontal cortex, which supports executive/attentional processes in working memory, has also been shown to play a role in organising information before it is stored in declarative memory (Fletcher et al., 1998).

Second, many – but not all – functions and tasks subserved by procedural memory can *also* be subserved by declarative memory, though generally in very different ways (Ullman, 2004). For example, such system redundancy has been found for route learning and navigation in humans and animals (e.g., hippocampal “place” learning in rodents, which relies on landmarks, *vs* striatal “response” learning, which relies on egocentric perceptual-motor skills) (Iaria et al., 2003; Packard, 2009), and in humans for learning and processing sequences, categories, and probabilistic rules (Fletcher et al., 2005; Foerde et al., 2006; Poldrack et al., 2001; Poldrack and Foerde, 2008; Willingham et al., 2002). Of interest here, such redundancy has also been proposed for grammar. Specifically, evidence has been forwarded to suggest that rule-governed complex forms and grammatical relations can rely not only on the procedural system, which learns the rules and combines forms into complex structures, but also at least partly – though likely not completely – on declarative memory, which can store complex forms as chunks, learn rules explicitly, or underlie conceptual/semantic parsing (Ullman, 2001, 2004, 2005, 2006a; Ullman and Pierpont, 2005). However, it is likely that not all aspects of grammar (or other functions) can be equally well subserved by either system; for example, long-distance dependencies in grammar may cause particular problems for declarative memory. Additionally, some functions and tasks can apparently be subserved *only* by one or the other system. For example, it appears to be the case that arbitrary associations, including for lexical knowledge, may always depend on declarative memory, while at least certain motor skills might require procedural memory (Dietrich et al., 2001; Ullman, 2004, 2005, 2006a, 2006b; Ullman and Pierpont, 2005).

Various factors affect whether a given function that can depend on either system (e.g., navigation, grammar) is actually learned or processed in one or the other (Poldrack et al., 2001; Poldrack and Rodriguez, 2004; Ullman, 2004). Of relevance here, a dysfunction of one system but not the other may result in an increased (compensatory) reliance on the intact system (Hartley and Burgess, 2005; Ullman, 2004, 2008). Thus, the impairment or attenuation of procedural memory has been shown to lead to an increased dependence on declarative memory for grammar and other functions. For example, in rats, navigation can be supported by the hippocampus following lesioning to structures that normally underlie procedural memory in this species (McDonald and White, 1995; Packard, 2008). In humans, a neuroimaging study of route learning found that individuals in the early stages of Huntington’s disease (which affects the basal ganglia) with mild symptoms showed basal ganglia activation, while those with severe symptoms showed hippocampal activation (Voermans et al., 2004). Moreover, disease severity did not correlate with participants’ route finding abilities, suggesting that the hippocampus compensated successfully for the basal ganglia impairments. Similarly, the dysfunction or attenuation of procedural memory in various situations and disorders, including in agrammatic aphasia (Drury and Ullman, 2002; Hagoort et al., 2003), autism (Walenski et al., 2006), and (see below) SLI (Ullman and Pierpont, 2005), have been found to lead to an increased dependence of grammar on declarative memory.

### 1.2 The Procedural Deficit Hypothesis (PDH) of SLI

Ullman and Pierpont (2005) proposed that the language problems in SLI can be largely explained by abnormalities of brain structures underlying procedural memory – in particular, portions of frontal/basal-ganglia circuits (especially the caudate nucleus and the region around Broca’s area) and the cerebellum. According to the PDH, these abnormalities should lead to impairments of the various domains and functions that depend on these structures. Most importantly, procedural memory itself is predicted to be impaired, leading to deficits in implicit sequence learning, grammar, and various other tasks and functions that depend on this system. Additionally, other, non-procedural, functions that depend at least in part on these structures should also tend to be problematic, including working memory. Unlike procedural memory deficits, which the PDH considers to be core deficits, impairments of other functions that depend on the same brain structures might or might not be observed, depending on the extent and nature of the underlying brain abnormalities (e.g., since different but parallel and anatomically proximate frontal/basal-ganglia circuits may underlie procedural and working memory) (Ullman, 2006a; Ullman and Pierpont, 2005). In contrast, the medial temporal lobe structures that underlie learning and consolidation in declarative memory are posited to remain largely normal, and thus declarative memory functioning should be essentially intact in SLI. Moreover, declarative memory is predicted to compensate, at least to some extent, for functions such as rule-governed aspects of grammar that are normally largely subserved by procedural memory, but that declarative memory can at least partly underlie.

Ullman and Pierpont (2005) accompanied their theoretical proposal with an in-depth review of the neural substrates of SLI, as well as of the status of language, memory, and other cognitive capacities in the disorder. Additionally, since the publication of their paper, a number of empirical studies examining these issues have been published. Overall, the data appear to largely support the pattern of predictions of the PDH. Here we briefly review those studies that are most relevant here.

All studies that have examined learning in procedural memory in SLI have observed deficits. These have been found both in the verbal domain in tasks that depend on procedural memory structures (Evans et al., 2009; Plante et al., 2002), and in non-verbal domains. Non-verbal procedural memory deficits, which we focus on in the present paper, have been observed both in probabilistic category learning (Kemény and Lukács, 2010) and implicit sequence learning (Lum et al., 2010; Tomblin et al., 2007). The sequence learning deficits have been examined with implicit visuo-spatial Serial Reaction Time (SRT) tasks, which have been independently shown to depend on procedural memory (Knopman and Nissen, 1991; Nissen and Bullemer, 1987; Siegert et al., 2006; Thomas et al., 2004). In a study by Tomblin et al. (2007), adolescents with SLI had slower learning rates of the sequence as compared to TD children. Lum et al. (2010) reported that children with SLI showed no sequence learning, whereas TD children did. Specifically, after repeated exposure to a visuo-spatial sequence, the response times of the TD children decreased, but then significantly increased when the visual stimulus was presented randomly (rather than as the sequence). This indicates the TD group learned aspects of the sequence. In contrast, no significant increase between sequenced and random blocks was observed for the SLI group. Additionally, a wide range of studies suggest that children with SLI have problems with motor skills, particularly with those involving sequences (Hill, 2001; Ullman and Pierpont, 2005). Finally, one of the hallmarks of SLI is impairments of grammar, especially of rule-governed aspects of grammar (Bishop, 1997; for a detailed review of language problems in SLI see Leonard, 1998; Rice et al., 1998; Rice et al., 1999; Ullman and Pierpont, 2005). Nevertheless, evidence suggests that declarative memory can at least partly compensate for these grammatical deficits in SLI, for example by storing complex forms as chunks, or learning explicit rules (Ullman and Pierpont, 2005).

Other, non-procedural, functions that depend in part on the implicated procedural memory system brain structures also seem to show impairments in SLI (Ullman and Pierpont, 2005). Of interest here are reports of working memory impairments in the disorder (for reviews see Gathercole and Alloway, 2006; Montgomery et al., 2010). Specifically, it has been found that children with SLI perform significantly more poorly on tasks requiring the short-term storage (Gathercole and Baddeley, 1990) and processing of verbal information (Archibald and Gathercole, 2006b; Ellis Weismer et al., 1999; Marton and Schwartz, 2003). In contrast, visuo-spatial working memory has generally been reported to be spared in SLI (Alloway et al., 2009; Archibald and Gathercole, 2006a, 2006b, 2007). The reasons for this contrast between impaired verbal working memory and largely normal visuo-spatial working memory are not yet clear (see Discussion).

The status of declarative memory in SLI has been examined in a limited number of studies. All studies that we are aware of have found normal learning in declarative memory for visual information (Baird et al., 2010; Bavin et al., 2005; Dewey and Wall, 1997; Lum et al., 2010; Riccio et al., 2007; Williams et al., 2000). These tasks have used a variety of paradigms that have been shown to depend on the declarative memory system (Lezak, 2004; Ullman et al., 2008). For example, dot learning tasks, in which participants are asked to remember a set of randomly placed dots (Cohen, 1997), and which have been found to be impaired in SLI (Riccio et al., 2007), appear to depend at least in part on right medial temporal lobe structures (Brown et al., 2010).

In contrast, the learning of verbal information in declarative memory has yielded a mixed pattern. (For simplicity, below we also refer to declarative memory for verbal information as verbal declarative memory, and likewise for visual declarative memory, and verbal and visuo-spatial working memory). Several studies have used list-learning paradigms. In this paradigm participants are typically presented with a list of words or word pairs, and are asked to orally recall the items immediately after each presentation, as well as following a short and/or long delay (Lezak, 2004). Considerable neuropsychological evidence indicates that such tasks are sensitive, at least in part, to left medial temporal lobe functioning (Davies et al., 1998; Lee et al., 2002; Ojemann and Dodrill, 1985). Studies of list-learning tasks have often found immediate recall to be impaired in SLI (Dewey and Wall, 1997; Lum et al., 2010; Nichols et al., 2004), though in other studies normal performance has been reported (Riccio et al., 2007; Shear et al., 1992). Delayed recall more often seems to be spared in SLI (Baird et al., 2010; Riccio et al., 2007; Shear et al., 1992), but sometimes shows impairments (Nichols et al., 2004). Studies have also been mixed with respect to delayed recognition for verbal information, alternatively reporting impaired (Nichols et al., 2004; Riccio et al., 2007; Shear et al., 1992) or normal (Baird et al., 2010) performance in the disorder. Story recall seems to result in impaired immediate recall, but largely normal performance after a delay (Baird et al., 2010; Merritt and Liles, 1987). Likewise, fast mapping tasks have yielded both deficits (Rice et al., 1990) and normal performance (Dollaghan, 1987). Importantly, most declarative memory task paradigms are subject to various confounds that may contribute to any observed deficits. In particular, at least the list and story learning paradigms depend heavily on working memory. Additionally, because verbal working memory tests involve language, the language deficits themselves in SLI could contribute to impaired performance on these tasks. However, neither working memory nor language deficits have been controlled for in any previous studies that we know of, and thus it remains unclear whether SLI is indeed associated with impairments of verbal declarative memory, once these factors are accounted for.

Finally, a number of studies have examined associations between measures of memory and language. To date, most research has focused on associations between measures of phonological short-term memory or working memory with tasks probing grammatical processing. In this literature, it has generally been found that non-word repetition tasks only weakly correlate with elicitation tasks assessing past tense knowledge (Bishop et al., 2006; Botting and Conti-Ramsden, 2001; Norbury et al., 2001). Correlations of a larger magnitude have been observed on tasks assessing phonological short-term memory or working memory with tasks of sentence comprehension (Montgomery, 1995, 1996, 2000, 2004; Montgomery and Evans, 2009). We are aware of only one study examining associations between language and declarative or procedural memory in SLI. In Tomblin et al. (2007), initially separate groups of adolescents with and without SLI were then re-organised into other groupings. In one analysis, all the participants (SLI and TD) were organised into two groups comprising those who scored either high or low on vocabulary tests. In a second analysis, two groups were formed based on whether they scored high or low on tests of grammatical ability. Group differences in the rate of learning on the SRT task were found between high and low grammar groups but not high and low vocabulary groups. These provide evidence linking grammatical (but not lexical) abilities to procedural memory, consistent with the PDH. However, declarative memory was not examined by Tomblin et al. (2007), and thus the relationship between this memory system and grammar, and whether declarative memory may play a compensatory role, remains unexplored.

### 1.3 The present study

In sum, previous studies have reported consistent deficits in SLI of verbal and non-verbal procedural memory. Working memory has yielded mixed results, with largely normal performance on visuo-spatial working memory tasks, but impairments of verbal working memory. Declarative memory has been found to be largely spared for visual information, but has yielded an inconsistent pattern of findings for verbal information.

However, a number of empirical gaps remain. First, little is known about the relative impairments of working, declarative and procedural memory, in particular in the same set of participants. Second, possible confounds such as language deficits (in verbal working memory and verbal declarative memory tasks) or working memory deficits (in various declarative memory tasks) have not been controlled for. Third, the relationship between the status of these memory systems on the one hand, in particular declarative and procedural memory, and lexical and grammatical abilities, on the other hand, let alone in the same set of children, remains largely unexplored.

The present study aims to fill these gaps. First, we examine performance on various measures of verbal and visual working, declarative and procedural memory systems in 51 children with SLI and 51 TD children. Second, we investigate the relationships between these memory measures and measures of grammatical and lexical abilities in both groups of children. Based on the PDH (Ullman and Pierpont, 2005), we tested the following predictions. SLI deficits are strongly predicted for procedural memory, even in a non-verbal domain. SLI deficits in working memory are likely. In contrast, children with SLI should be largely spared at declarative memory, even in the verbal domain, once working memory and language deficits are controlled for. Associations between memory and language measures should yield correlations between declarative memory and lexical abilities in both SLI and TD children (since all individuals must depend on declarative memory for lexical knowledge; see above). In TD children, grammatical abilities are expected to correlate with procedural memory. Children with SLI should show the same correlation, and/or grammatical abilities should correlate with declarative memory, given its predicted compensatory role.

## 2 Methods

### 2.1 Participants

Fifty-one primary school aged children with SLI (35 males, 16 females) and 51 TD children (35 males, 16 females) of comparable age and non-verbal ability participated in the study (Table 1). All children were recruited from the northwest of England, and all came from homes where English was spoken as the first language. The children with SLI obtained a Core Language Score (CLS) of −1.25 SD or less on the Clinical Evaluation of Language Fundamentals-4th Edition, UK Standardisation (CELF-4 UK, Semel et al., 2003), and a Performance IQ (PIQ) score no less than 1 SD below the mean on the Wechsler Abbreviated Scale of Intelligence (WASI, Wechsler, 1999). TD children obtained standardised scores within one standard deviation (SD) of the mean on both the CELF-4 UK and WASI. The SLI and TD groups differed on the CLS and CELF (Expressive Language Index – ELI, Receptive Language Index – RLI) language measures, but not on age or PIQ.

### 2.2 Materials

Working memory, declarative memory, procedural memory and lexical and grammatical abilities were all assessed with well-studied measures of these domains.

#### 2.2.1 Working memory

Working memory functioning was assessed with the Working Memory Test Battery for Children (WMTB-C, Pickering and Gathercole, 2001). This test comprises eight subtests, which were designed to assess the central executive, phonological loop, and visuo-spatial sketchpad components of Baddeley’s (2003) model of working memory (for validation study see Gathercole et al., 2004). All subtests from the WMTB-C are standardised to a mean of 100 and SD of 15.

The central executive component is assessed by the Listening Recall, Counting Recall, and Backward Digits Recall subtests, all of which require the short-term storage and processing of information. On Listening Recall, children are presented with a series of sentences. For each sentence, they must first provide true/false judgements on the sentence’s semantics, and then recall the sentence-final word. The Listening Recall subtest is an adaptation of the Competing Language Task (Gaulin and Campbell, 1994). On the Counting Recall task, children are presented with pictures of randomly presented dots, and are asked to count and then recall the dots. Counting Recall is based on the counting span task developed by Case et al. (1982). The Backward Digit Recall subtest, in which children are asked to repeat a string of digits in reverse order, is similar to the Backward Digit Span Task in the Wechsler Intelligence Scales (e.g., Wechsler, 2003, 2008). These subtests are likely to probe not only Baddeley’s central executive, but also Cowan’s focus of attention. Note that while all these subtests are designed to measure central executive (and likely attentional) working memory functioning, as they require both the short-term storage and processing of information, it is important to emphasise that all have a verbal component, and thus likely depend more generally on verbal aspects of working memory. The WMTB-C does not include central executive tasks which can be considered non-verbal.

The phonological loop was assessed with four subtests: Digit Recall, Word List Matching, Word List Recall, and Non-word List Recall. On these subtests children were asked to temporarily store and then recall digits, words or non-words. The visuo-spatial sketchpad was evaluated by the Mazes Memory and Block Recall subtests. Both subtests require children to temporarily store visual information. On Mazes Memory, children are first shown a picture of a completed maze for 3 sec, with the solution showing how to exit the maze shown in red. They are then presented with a non-completed version of the same maze and asked to draw a facsimile of the solution. The Block Recall subtest is an adaptation of the Corsi Blocks test (Corsi, 1972). Children are seated in front of an array of randomly placed blocks. The test administrator taps on the blocks and children are asked to then tap the blocks in the same order.

#### 2.2.2 Declarative memory

The Children’s Memory Scales (CMS, Cohen, 1997) provides measures that quantify aspects of the learning and retrieval of verbal and non-verbal information in declarative memory. The CMS is similar to the Wechsler Memory Scale-3rd Edition (Wechsler, 1997), and shares nearly all its declarative memory subtests. In the present study, only the declarative memory CMS subtests were presented to the children, since working memory was measured with the WMTB-C. Considerable neuropsychological evidence suggests that the CMS subtests designed to probe declarative memory indeed assess (as well the WMS-III) the neural structures that support this memory system (Brown et al., 2010; Cohen, 1997; Jambaqué et al., 2007; Ojemann and Dodrill, 1985).

Learning and retrieval of verbal information was assessed with the Word Pairs and Stories subtests. On Word Pairs, children are presented with a list of 14 semantically unrelated word pairs (e.g., rice-chair). Subsequently, the first word in each pair is provided, and the child must recall the second (Learning). The children are then asked to recall both words in all pairs (Short Recall). After other subtests on the CMS have been administered (typically about 30 min), children are again asked to recall the full list of word pairs (Delayed Recall). This is followed by the presentation of the 14 word pairs along with 14 distracter pairs, with the children indicating whether or not they recognise the target pairs from earlier in the test (Delayed Recognition). On the Stories subtest, children are presented with two stories of equal length, which they are asked to recall verbatim following the presentation of each (Short Recall). Scores are based on the number of words and themes that were correctly recalled. After a delay in which other tests are given (typically about 35 min), Delayed Recall and then Delayed Recognition of both words and themes are assessed. Aspects of the learning and retrieval of visual information were assessed by the Dot Locations and Faces subtests. These subtests have a similar structure to the verbal subtests. Results from all CMS core subtests used are reported in this study. Each measure is standardised to a mean of 10 and SD of 3.

#### 2.2.3 Procedural memory

Procedural memory was assessed using a version of Nissen and Bullemer’s (1987) SRT Task. This task is designed to test implicit visuo-spatial sequence learning in procedural memory. In SRT tasks, participants are typically asked to press one of four response buttons, each of which matches the location of a visual stimulus presented on a computer monitor. Unbeknownst to participants, the visual stimulus follows a predefined sequence. After multiple exposures to the sequence, a random pattern of visual stimuli (rather than the predefined sequence) is presented. In neurologically intact children and adults, reaction times (RTs), which are the principal dependent measure of interest in SRT tasks, typically decrease during the repeated presentation of the sequence, and increase from the final sequence presentations to the random patterns (e.g., Nissen and Bullemer, 1987; Thomas et al., 2004). This RT increase is taken as evidence that knowledge of the sequence has been learned. To determine whether the knowledge is purely implicit, explicit knowledge of the sequence is probed. Substantial neuroimaging and neurological evidence suggests that implicit sequence learning in SRT depends on the procedural memory system (Knopman and Nissen, 1991; Siegert et al., 2006; Thomas et al., 2004). For example, patients with neural pathology affecting the basal ganglia and cerebellum perform more poorly on implicit sequence learning than control groups, with the sequence-to-random increase either missing or decreased as compared to controls (Knopman and Nissen, 1991; Nissen, 1992; Nissen and Bullemer, 1987; Nissen et al., 1989; Siegert et al., 2006).

Note that in the current study, unlike working and declarative memory, no verbal or auditory analogue of this task was given to participants. This was, first of all, because auditory SRT tasks require participants to discriminate between tones of different frequencies (e.g., Zhuang et al., 1998), which might be problematic for children with SLI (Hill et al., 2005; McArthur and Bishop, 2004). Additionally, our focus on a visuo-spatial SRT task was not considered to be problematic for testing the PDH, since, as we have seen above, the classic (and much more widely studied) visuo-spatial version of this task has been shown to depend on procedural memory structures, including those structures implicated by Ullman and Pierpont (2005).

In the SRT Task used here, children were seated in front of a computer monitor, on which a visual stimulus (a yellow smiley face) repeatedly appeared in one of four horizontally arranged spatial locations. The children were instructed to press one of four horizontally arranged buttons (on a response box) that corresponded to each of the four locations on the screen. Presentation of the visual stimulus was divided into five blocks, each comprising 90 stimulus presentations. In blocks 1 through 4 the location of the visual stimulus adhered to a 10-item sequence pattern identical to that used by Nissen and Bullemer (1987). On the 5th block the stimulus appeared randomly, with the following constraints: the stimulus appeared in each spatial location an equal number of times, and with an equal probability of transitions, as in the sequence blocks. After the 5th block had been completed, explicit knowledge of the sequence was assessed by asking children to recall the pattern. There were four recall trials. At the start of each trial the visual stimulus appeared. For Trial 1 in the first position of the sequence, for Trial 2 in the second position, for Trial 3 in the third position and for Trial 4 in the fourth position. Children were then asked to point to the next nine locations they thought the visual stimulus would appear. We took a liberal approach by counting as correct any correct response even if any prior positions were incorrect. Using this approach, on none of the recall trials were either the SLI or TD children significantly above chance (i.e., above 2.5), nor did they differ significantly from each other.

Children’s accuracy and RTs were both recorded. To control for within-subject variability in motor speed, each child’s RTs were converted to *z*-scores referenced to the median and SD across all correct trials for that child. Normalising data in this way effectively ensured that all children’s shortest RTs have approximately the same value, and similarly for their longest RTs. For example, if the longest RT for one child was 5000 msec and longest for another was 1000 msec, after *z*-normalising the values for both children might be 5 (i.e., 5 SD above the median of their overall RTs). This approach has been previously used to examine differences between children and adults on SRT tasks (e.g., Thomas et al., 2004). Finally, we also addressed potential attention lapses in this task. This was considered important since the task was long, with five blocks each of 90 trials (about 13 min). To deal with this concern, we deleted data points for each child whose RTs were 3 SD or more above his/her mean RT. The average mean number of data points deleted per child was 9.29 (SD = 3.087, Range: 1–17) for the TD group, and 9.35 (SD = 3.827, Range: 1–15) for the SLI group. This difference was not statistically significant [*t* (100) = .076, *p* = .940]. Thus removal of outliers did not significantly differentially affect one group.

#### 2.2.4 Lexical abilities

Children’s lexical abilities were assessed with the Expressive One-Word Picture Vocabulary Test (EOWPVT, Brownell, 2000a) and the Receptive One-Word Picture Vocabulary Test (ROWPVT, Brownell, 2000b). In the EOWPVT children are asked to name a presented picture. In the ROWPVT children are shown four pictures, and are asked to point to the one of four pictures that matches an orally presented target word. Each test comprises 170 items. Testing is discontinued if the child makes six errors within eight consecutive items. A score of one is awarded for each correct answer. The use of both expressive and receptive vocabulary tests allowed us to obtain a measure of lexical knowledge that was comparable to the composite measure of lexical knowledge used by Tomblin et al. (2007).

#### 2.2.5 Grammatical abilities

Expressive grammatical abilities were assessed with the Grammar subscale from the Action Picture Test (Renfrew, 1988), and receptive grammatical abilities with the Test for Reception of Grammar 2nd Edition (TROG-2, Bishop, 2003). In the Action Picture Test, children are shown pictures, and are asked a question about each one. Children’s responses are recorded and scored with respect to the use of grammar. There are a total of 10 pictures; the highest possible raw score is 36. The TROG-2 consists of 80 sentences evenly divided into 20 blocks. Children are presented with a sentence and asked to point to the matching picture from four possible options. As children progress through each block, increasingly more complicated syntactic structures are presented. A child does not pass a block if s/he failed at least one item. Testing is discontinued if the child fails five consecutive blocks. The data used in the analyses were the total number of blocks passed. As with lexical knowledge, the use of both expressive and receptive measures of grammatical knowledge allowed for our measure to be comparable to the one used by Tomblin et al. (2007).

### 2.3 Procedure

The test battery was administered to participants over five sessions, all of which took place within a 3-month period. Only one memory task was presented per session. The order of presentation of tasks was randomised across participants. Ethical approval for the study was obtained from The University of Manchester, and informed written consent was gained from the children’s parents or legal guardians.

## 3 Results

### 3.1 Lexical and grammatical abilities

Summary statistics are presented in Table 2. The SLI group performed significantly worse than the TD group on all four lexical and grammatical measures. All comparisons yielded large effect sizes.

### 3.2 Working memory

Potential group differences in working memory were examined on the subtests of the WMTB-C. Between-subjects MANOVAs (Table 3, Covariates: None) revealed a significant multivariate group effect for the working memory subtests designed to probe the central executive (*p* < .001), and for those assessing the phonological loop (*p* < .001), both of which showed large effect sizes (*partial η*^2^ ≥ .138, Cohen, 1988). In contrast, the multivariate group effect for the subtests probing the visuo-spatial sketchpad was not significant (*p* = .179), and yielded a small (i.e., *partial η*^2^ < .059) effect size. Univariate post-hoc tests were then performed to examine potential group differences on each working memory subtest (Table 4, under the column “No covariates”). For all univariate post-hoc analyses (here and elsewhere), alpha was adjusted using Holm’s Procedure to control for multiple comparisons (Aicken and Gensler, 1996; Holm, 1979). The univariate tests revealed significant group differences, and mostly large effect sizes, on all central executive and phonological loop subtests, whereas no significant differences and small effect sizes were observed on the two subtests that assessed the visuo-spatial sketchpad.

Because the subtests designed to probe the central executive and phonological loop depend heavily on language, it is possible that the observed working memory deficits in the participants with SLI might be due to their language problems rather than to working memory deficits per se. Therefore we performed additional analyses in which we covaried out a measure of language abilities. We computed a single composite variable of language by submitting the four measures of language (expressive and receptive lexical and grammatical abilities; see Table 2) to a principal components analysis, and extracted a single factor. This approach aims to create a composite variable that maximizes the shared variance of all four language measures, and minimizes the variability that is unique to a single measure or is shared only between two or three of them. The four measures accounted for 67.7% of the variance in the language factor. The factor loadings were as follows: Expressive Vocabulary = .853, Receptive Vocabulary = .832, Expressive Language = .769 and Receptive Grammar = .834. The MANCOVAs with the language factor included as covariate yielded significant multivariate group effects both for the central executive (*p* < .001) and phonological loop subtests (*p* < .001), although with a reduction of effect sizes in both cases (Table 3, Covariates: Language Factor). The post-hoc univariate tests controlling for language abilities revealed significant differences on all the central executive and phonological loop subtests except the Word List Matching subtest, mostly with medium (*partial η*^2^ ≥ .059) or large effect sizes (Table 4, under “Covariate: Language Factor”).

### 3.3 Declarative memory

The next set of analyses tested SLI-TD group differences on the CMS, to examine declarative memory for verbal and visual information. Results from between-subjects MANOVAs revealed a significant multivariate group effect for the subtests probing verbal information (*p* < .001), with a large effect size, but not for the subtests of visual information (*p* = .350), which yielded a small effect size (Table 3, Covariates: None). The post-hoc univariate tests (Table 5, under “No covariates”) yielded significant group differences, with medium to large effect sizes, on all measures designed to assess verbal aspects of declarative memory. In contrast, small effect sizes were found on all visual subtests, only one of which showed a significant group difference.

Many of the subtests from the CMS require children to temporarily store information, and thus the observed group differences could in part be explained by working memory deficits rather than problems with declarative memory itself. Group differences on the CMS were therefore examined while controlling for working memory. Three composite scores were computed for each subject – for the central executive, phonological loop, and visuo-spatial sketchpad subtests – by summing the *z*-scores of the subtests designed to assess each of these aspects of working memory. These three composite scores were then entered as covariates into separate MANCOVAs for verbal and visual declarative memory (Table 3, Covariate: Working Memory). These analyses revealed, first of all, a statistically significant multivariate group effect for the declarative memory subtests of verbal information (*p* = .009), though with a smaller effect size than the analogous model with no covariates. The MANCOVA on the declarative memory subtests of visual information revealed no group differences (*p* = .278). The univariate tests examining group differences while controlling for working memory (Table 5, under “Covariates: Working Memory”) yielded mostly small or medium effect sizes for the verbal information subtests, with only two of the subtests showing significant group differences (Short and Delayed recall of the Stories subtest). None of the visual information subtests yielded significant univariate group differences, and all showed small effect sizes.

As with the working memory subtests that involve language, any observed SLI deficits on the verbal declarative memory subtests could be due to language problems rather than impairments with declarative memory itself. Therefore we analysed the verbal declarative memory subtests while covarying the language factor described above. The MANCOVA yielded a significant multivariate group effect (*p* = .042), though with a further reduction (to medium) of the effect size (Table 3, Covariates: Language Factor). Controlling for language abilities, none of the univariate analyses of the individual measures of verbal declarative memory were significant, and all showed small to medium effect sizes (Table 5, under “Covariate: Language Factor”).

Finally, to remove confounds of both working memory and language in the declarative memory subtests of verbal information, we included the three working memory composite scores as well as the language factor as covariates in the analyses. The multivariate group effect was not significant (*p* = .328, Table 3). Moreover, none of the univariate group differences (Table 4, under “Covariates: Working Memory & Language Factor”) were significant, and all showed small effect sizes.

### 3.4 Procedural memory

We investigated procedural memory by examining sequence learning with the SRT task. We first probed accuracy. The average proportion of correct responses for both groups approached ceiling (SLI: *M* = .89, SD = .08, Min = .69, Max = .99; TD: *M* = .92, SD = .06, Min = .62, Max = .99). An independent samples *t*-test on arcsine transformed proportions, to correct for non-normality, revealed no significant group difference in accuracy [*t*(100) = 1.681, *p* = .096, *partial η*^2^ = .027]. These results suggest that the two groups were responding with comparable levels of accuracy.

**Fig. 1:**
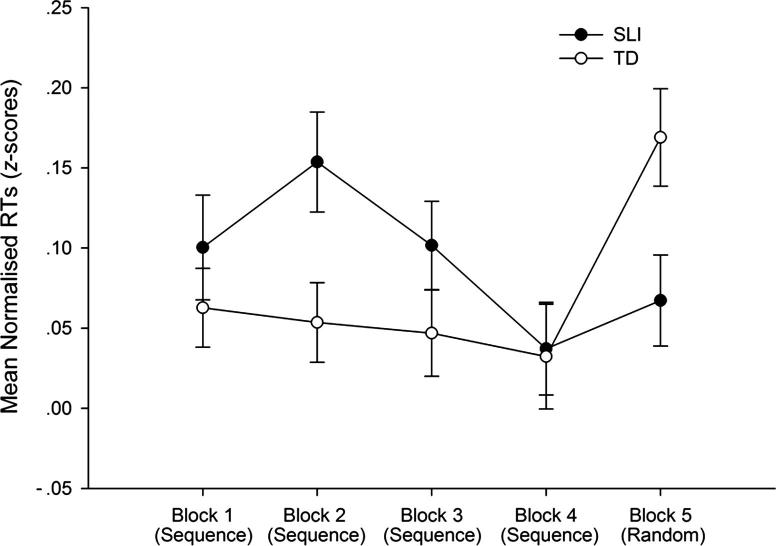
Mean normalised RTs reported by Block and Group (error bars show standard error).

We next focused on RTs, which constituted the primary dependent measure. Mean normalised RTs for correct responses reported by block for each group are presented in Fig. 1. Analyses examined SLI-TD group differences in the RT difference between block 4 (sequence pattern) and block 5 (random pattern). The dependent measure was computed as the difference in normalised RTs between blocks 4 and 5 (Thomas et al., 2004). One-way repeated-measures ANOVA revealed a significant effect of group [*F*(1,102) = 5.17, *p* = .026, *partial η*^2^ = .058], with an approximately medium effect size, indicating a larger RT difference between blocks 4 and 5 for the TD children than the children with SLI. Moreover, one-way ANOVAs showed that the change in (normalised) RTs between blocks 4 and 5 was statistically significant (after correction for multiple comparisons) for the TD group [*F*(1,49) = 10.864, *p* = .004, *partial η*^2^ = .194], with a large effect size, but not the SLI group [*F*(1,49) = 1.118, *p* = .520, *partial η*^2^ = .029]. This indicates that the TD group but not the SLI group showed significant sequence learning.

Finally, we performed additional analyses with the three composite scores of working memory covaried out, to test whether any dependence of the task on working memory might explain the observed SLI deficit. The one-way ANCOVA yielded significant group differences [*F*(1,99) = 4.56, *p* = .038, *partial η*^2^ = .052], with a small effect size, due to a greater RT difference between blocks 4 and 5 for the TD than SLI children. We did not perform within-subject comparisons of blocks 4 and 5 (i.e., within the TD and SLI children) because the correlations between the three working memory covariates and the dependent RT variables (block 4, block 5, block 4–5 difference) were not significantly different from zero for either the TD children (Range of Pearson’s *r* values: −.038 to .143, all n.s. different from zero) or the children with SLI (−.207 to .275, again all n.s.). That is, working memory was not significantly correlated with performance on the SRT task within each group. Thus, the SLI deficit at procedural learning was not explained by working memory impairments.

### 3.5 Relationships between memory and language measures

The next set of analyses examined the relationship between the different memory (sub)systems on the one hand, and grammatical and lexical abilities on the other. For working memory, we used the three composite scores described above, that is, composites for the subtests designed to assess the central executive, phonological loop, and visuo-spatial sketchpad. For declarative memory, we computed analogous composite measures: one from the *z*-scores of the verbal declarative memory subtests, and another for the visual declarative memory subtests. For procedural memory, we used the difference scores between blocks 4 and 5 described above. For lexical abilities, we computed a composite score by summing the *z*-scores of the expressive (EOWPVT) and receptive (ROWPVT) tests. Likewise, for grammatical abilities, we computed a composite score from the *z*-scores of the expressive (Action Picture Test) and receptive (TROG-2) tests of grammar (after applying a reflected square root transformation to the raw TROG-2 scores to correct for a skewed distribution). Associations between the memory and language variables were examined with correlations (Pearson’s *r*) computed separately for each pair of memory (central executive, phonological loop, visuo-spatial sketchpad, verbal declarative memory, visual declarative memory, procedural memory) and language (lexical abilities, grammatical abilities) measure being examined, separately for the TD and SLI groups.

None of the three working memory measures (for the central executive, phonological loop, visuo-spatial sketchpad) correlated significantly with either lexical or grammatical abilities in either the TD or SLI groups (Table 6). In contrast, lexical abilities correlated with verbal declarative memory, with large effect sizes (i.e., Pearson's *r* ≥ .371, Cohen, 1988), in both the TD and SLI groups (Table 6). Lexical abilities were not correlated with visual declarative memory, which yielded small to medium effect sizes. However, a direct comparison of the *r*-values for the correlations of lexical abilities with verbal and visual declarative memory revealed no significant differences between them, either for the TD group [*t*(48) = 1.51, *p* = .139] or the SLI group [*t*(48) = 1.05, *p* = .298]. Grammatical abilities showed a different pattern. These were correlated with procedural memory for the TD group and verbal declarative memory for the SLI group (Table 6). A direct comparison of the *r*-values for the correlations of grammatical abilities with verbal and visual declarative memory in SLI yielded a borderline significant difference between the two [*t*(48) = 1.61, *p* = .057].

Finally, we examined whether the observed pattern of correlations could be explained by working memory. First, we tested whether any of the three working memory composites (for the central executive, phonological loop, and visuo-spatial sketchpad) correlated with either any of the declarative memory, procedural memory, lexical, or grammatical measures. Only the central executive composite correlated with visual declarative memory for the TD children and with verbal declarative memory for the SLI children. However, even after controlling for the influence of the central executive on visual declarative memory in the TD children, and on verbal declarative memory in the SLI children, the correlations showed the same pattern as described above. Therefore working memory did not explain the pattern of correlations between language and declarative or procedural memory.

## 4 Discussion

This study examined multiple measures of working, declarative, and procedural memory in native English-speaking children with and without SLI of about 10 years of age. The children with SLI were impaired at a visuo-spatial procedural memory task, even when controlling for working memory. In contrast, they showed normal performance at visual declarative memory, and at verbal declarative memory once working memory and language deficits were controlled for. Working memory showed a mixed profile. Verbal short-term memory (assessed by subtests designed to probe the putative phonological loop) and verbal working memory (assessed by verbal central executive/attentional working memory subtests) were impaired, even when controlling for language deficits. In contrast, the short-term storage of visual information was spared.

Correlation analyses between memory and language measures revealed the following. Working memory did not correlate with language: none of the measures assessing the different components of working memory (verbal short-term memory, verbal working memory, visual short-term memory) correlated significantly with either lexical or grammatical abilities in either SLI or TD children. In contrast, declarative memory, in particular verbal declarative memory, correlated with lexical abilities in both groups of children. Finally, grammatical abilities were associated with procedural memory in the TD children, but with verbal (and not visual) declarative memory in the children with SLI.

The results suggest the following. Children with SLI have a deficit in procedural memory, even in a non-verbal domain. Declarative memory appears to be spared, both in the visual domain, and in the verbal domain once working memory and language deficits are accounted for. Working memory is normal in the visual domain, but not in the verbal domain. In both TD and SLI children, lexical abilities are related to declarative memory. In TD children, grammatical abilities are associated significantly with procedural memory, but not declarative memory. In children with SLI, in contrast, grammar is associated significantly with declarative memory, but not procedural memory.

These findings are largely consistent with the PDH, which this study was designed to test (Ullman, 2004; Ullman and Pierpont, 2005). First and foremost, the observed deficits in procedural memory support the primary (core) prediction of the PDH, that procedural memory is impaired. The results are consistent with previous studies, all of which have also reported impairments at learning in procedural memory, in both verbal and non-verbal domains (see Introduction).

The PDH also predicts that working memory impairments may be found in SLI. These are not considered core deficits in the disorder, but are nonetheless likely. The present study replicates previous findings that the short-term storage and processing of verbal information (i.e., verbal short-term and working memory) are impaired in SLI (Introduction), and shows for the first time that these deficits hold even when language problems are held constant. The finding that visual working memory remains spared is also consistent with previous studies (see, Introduction). Overall, the pattern of results in this and other studies indicates that visual working memory tends to remain normal in SLI. It is not clear why verbal working memory is impaired, even when language deficits are controlled for, while visual working memory remains normal. One possibility is that poor visuo-spatial memory skills are found only in a subgroup of children with SLI (Archibald and Gathercole, 2006a). Another possibility is that working memory itself is actually largely normal in SLI, and that problems in verbal working memory are due to the language deficits in the disorder (Alloway et al., 2009). In the current study, verbal working memory deficits remained, though with reduced effect sizes, once the language composite was covaried out. However, it is possible that controlling for other language measures (e.g., of phonology) might further reduce or eliminate the observed verbal working memory deficit. Further studies seem warranted to elucidate the apparent dichotomy between impaired verbal but normal visual working memory in SLI.

The PDH expects declarative memory to remain largely normal in the disorder. The finding that children with SLI were spared not only at visual declarative memory, but also at verbal declarative memory once working memory and language deficits were accounted for, supports this prediction. The sparing of visual aspects of declarative memory is consistent with previous studies (see, Introduction). Together, this and other studies suggest that visual declarative memory remains largely intact in SLI. As we have seen, previous studies of verbal declarative memory have reported a mixed pattern of results in SLI. In particular, immediate recall in list or story learning paradigms has generally been found to be impaired, while performance after a delay is inconsistent across studies. Based on the results of the current study, we hypothesise that previous inconsistent findings in SLI research with respect to delayed memory measures, and indeed declarative memory in general, might reflect at least in part individual or task differences in demands placed on working memory and language. Indeed, in this study, after holding these two variables constant, no SLI impairments in verbal declarative memory were observed. This pattern of results is consistent with a profile of some working memory impairments, but with spared declarative memory, even in the verbal domain (Ullman and Pierpont, 2005).

The correlations between declarative memory and lexical abilities in both the TD and SLI children support the predictions of the PDH, and of the declarative/procedural (DP) theory more generally, that lexical memory depends on declarative memory, and that simple (underived) words must always be learned in this system (Ullman, 2001, 2004, 2007; Ullman and Pierpont, 2005). The finding that lexical abilities correlated significantly only with verbal declarative memory, but that this correlation did not differ from the correlation between lexical abilities and visual declarative memory, suggests a primacy for verbal declarative memory in lexical memory, but a role for visual aspects of declarative memory as well. A lexical role even for visual declarative memory is not surprising, given that much of the conceptual knowledge associated with words can also depend on visual information. Note that the apparent lexical role of visual declarative memory observed here does not appear to be due simply to task effects, that is, to the presence of pictures in the lexical tasks: pictures were also critical in the grammatical tasks (see Materials), yet grammatical abilities did not correlate at all with visual declarative memory, in either the SLI or TD children (Table 6).

The correlation between procedural memory and grammatical abilities in TD children also supports the predictions of the PDH and the DP theory – specifically, that in cognitively intact individuals aspects of grammar are learned in and processed by the procedural memory system. The correlation between declarative memory and grammatical abilities in SLI children supports the predictions of the PDH that declarative memory should tend to compensate for impaired procedural memory in SLI by taking over aspects of grammar. Note that the PDH expects that grammar should *also* correlate with procedural memory in SLI, since deficits in procedural memory are posited to explain most of the grammatical problems in the disorder. Indeed, this pattern was observed by Tomblin et al. (2007). The pattern observed here suggests that declarative memory may have played a more important compensatory role for the tested grammatical abilities in these children with SLI, leaving little variability in grammatical abilities to be explained by the observed procedural memory deficits. Interestingly, the significant correlation in SLI between grammatical abilities and verbal declarative memory did not differ significantly from the (non-significant) correlation in SLI between grammatical abilities and procedural memory [*t*(48) = .97, *p* = .33]. This suggests that procedural memory indeed played some role in these grammatical abilities in the children with SLI. Additionally, the analogous comparison for the TD children was also not significant [*t*(48) = .39, *p* = .70], consistent with the hypothesis that even in healthy individuals declarative as well as procedural memory play roles in rule-governed aspects of grammar (Ullman, 2004; Ullman, 2007). Finally, the finding that verbal but not visual declarative memory was associated with grammatical abilities in SLI and TD children suggests that only verbal aspects of declarative memory play a role in grammar. This is indeed not surprising, given that grammar (unlike lexical knowledge) does not seem to rely on visual information.

The lack of significant correlations between lexical or grammatical abilities on the one hand, and verbal or visual working memory (including measures of the putative central executive component) on the other, suggests that the lexical and grammatical problems in SLI are not strongly related to working memory impairments. This finding is consistent with previous studies, which have often reported small and non-significant correlations between working memory and grammar measures in SLI (see, Introduction). The results throw further doubt on strong versions of claims that working memory deficits alone can fully account for normal language development (Baddeley et al., 1998) and for the language impairments in SLI (Gathercole and Baddeley, 1990).

It might be argued that an absence of a correlation between working memory and grammar (or indeed the potential absence of clear and consistent working memory impairments, as discussed above), contradicts the PDH (Bishop et al., 2006). However, the PDH claims that the primary, core, deficit in SLI is of procedural memory, which is mainly responsible for the grammatical impairments in the disorder. Working memory and other non-procedural functions that depend in part on the affected brain structures underlying procedural memory are expected to co-occur probabilistically with these core deficits. The likelihood of such co-occurrence depends on factors such as the anatomical proximity of those portions of the affected structures (e.g., frontal/basal-ganglia circuits) responsible for these functions to those portions that underlie procedural memory (and in particular, to those portions that underlie those aspects of procedural memory that subserve grammar) (Ullman and Pierpont, 2005). Indeed, as we have seen above (see, Introduction), procedural memory seems to depend more on BA 44 and premotor frontal regions, and working memory more on other prefrontal areas, including BA 46 and BA 45/47. Thus, although the PDH expects that the neural abnormalities underlying procedural memory may often extend to these frontal regions subserving working memory (and the portions of the basal ganglia they are connected to), such abnormalities, and their accompanying functional deficits of working memory, are not expected to be a core feature of the disorder, and are unlikely to constitute the primary cause of the language problems in SLI (Ullman, 2004, 2006a; Ullman and Pierpont, 2005).

The findings reported here may also help inform other explanatory hypotheses of SLI. The observed memory deficits, in particular of visuo-spatial procedural memory, contradict strong versions of hypotheses that posit that only deficits of language, in particular of grammar, occur in SLI (Rice, 2000; van der Lely, 2005). The correlation between declarative memory and grammatical abilities in SLI is also problematic for such hypotheses. Additionally, this correlation is not expected on the view that the language problems in SLI are explained by phonological deficits (Joanisse, 2004). Similarly, this correlation, together with the lack of a correlation between either lexical or grammatical abilities in SLI with any working memory measures, does not appear to be predicted by accounts that posit that the language deficits in SLI are caused by processing deficits (Leonard, 1998; Tallal, 2004).

This study has various limitations that may be addressed by future studies. Although we examined verbal and non-verbal measures of working memory and declarative memory, only a non-verbal measure of procedural memory was included. On the one hand, this is sufficient for testing the PDH, which expects that even non-verbal procedural memory deficits should be observed in SLI. And given that any verbal procedural memory measure may be contaminated by language deficits, this is a purer approach. Nevertheless, future studies examining the status of working, declarative and procedural memory in SLI would benefit from the inclusion of measures of verbal procedural memory as well. The present study also leaves many other avenues open for further research. We did not examine *how* declarative memory may underlie grammar in its compensatory role – e.g., via chunking, learning rules explicitly, or conceptual/semantic parsing (see, Introduction). Additionally, although the present study tested associations between performance at memory systems and lexical and grammatical abilities, it did not investigate any causal effects of the posited dependence of these abilities on declarative or procedural memory. Finally, we limited our investigation to behaviour, and did not probe the neural bases of SLI, or of the observed language and memory deficits in the disorder.

In conclusion, the evidence from this and other studies seems to suggest the following. SLI is associated with procedural memory deficits. Declarative memory is intact for visual information, and for verbal information once working memory and language deficits are controlled for. Working memory is normal for visuo-spatial information, but appears to be problematic in the verbal domain. Lexical abilities in SLI (and TD) children are related at least in part to declarative memory. In TD children, grammatical abilities are related at least partly to procedural memory. In SLI, variability in grammatical abilities seems to be explained both by procedural memory deficits and by compensation by the largely intact declarative memory system. Overall, the evidence appears to largely support the predictions of the Procedural Deficit Hypothesis, or PDH (Ullman and Pierpont, 2005), though additional research is needed to further investigate a number of issues. In sum, this study highlights the importance of simultaneously considering multiple memory systems and their interactions in developing our understanding of the nature of the language difficulties in SLI.

## Figures and Tables

**Table 1:** Age and standardised tests: summary scores and comparisons.

Variable	SLI (*n* = 51)			TD (*n* = 51)			Comparison		
*M*	SD	Range	*M*	SD	Range	*t*	*p*	
Age (months)	117.6	8.9	103–137	118.2	8.5	102–137	.37	.714	.001
CLS	71.3	8.7	48–82	99.4	6.1	90–114	18.83	<.001	.777
ELI	71.1	9.6	49–87	98.9	7.0	83–114	16.75	<.001	.734
RLI	75.7	7.6	58–88	98.5	8.7	83–119	14.14	<.001	.661
PIQ	98.0	7.3	85–112	99.6	7.6	85–115	1.13	.260	.007

Note: age expressed in months. CLS, ELI, RLI and PIQ have a mean of 100 and SD of 15. Abbreviations: CLS = Core Language Score, ELI = Expressive Language Index, RLI = Receptive Language Index, PIQ = Performance IQ.

**Table 2:** Lexical and grammatical abilities: summary scores and comparisons.

Measure	SLI			TD			Comparison		
*M*	SD	Range	*M*	SD	Range	*t*	*p*	*partial η*^2^
*Lexical abilities*									
EOWPVT (expressive vocabulary)	82.3	13.2	54–107	99.2	9.1	81–124	7.527	<.001	.362
ROWPVT (receptive vocabulary)	94.3	13.0	65–128	105.3	10.6	78–130	4.673	<.001	.179
*Grammatical abilities*									
Action Picture Test (expressive grammar)	24.8	5.4	13–35	29.2	4.1	19–36	4.627	<.001	.176
TROG-2 (receptive grammar)	11.8	3.5	3–18	16.5	2.5	9–20	7.859	<.001	.382

**Table 3:** MANOVAs and MANCOVAs examining SLI-TD group differences on working memory and declarative memory.

Memory system/dependent variables	Covariates	Hotellings trace	*F*	*p*	*partial η*^2^
*Working memory*					
Central executive tests	None	.643	21.020	<.001	.392
Language factor	.234	9.893	<.001	.234
Phonological loops tests	None	.594	14.410	<.001	.373
Language factor	.382	9.178	<.001	.277
Visuo-spatial sketchpad Tests	None	.035	1.750	.179	.034
*Declarative memory*					
Verbal information tests	None	.575	7.726	<.001	.365
Working memory	.221	2.873	.009	.181
Language factor	.165	2.195	.042	.131
Working memory & language factor	.091	1.171	.328	.083
Visual information tests	None	.059	1.129	.350	.056
Working memory	.069	1.284	.278	.065

**Table 4:** Working memory: WMTB-C summary scores and comparisons.

Variable	SLI			TD			Effect size (*partial η*^2^)	
*M*	SD	Range	*M*	SD	Range	No covariates	Covariate: language factor
*Central executive*								
Listening Recall subtest	87.5	15.9	57–117	104.3	13.1	68–133	.255∗∗	.097∗
Counting Recall subtest	80.0	19.0	8–110	99.4	10.4	67–121	.291∗∗	.199∗∗
Backward Digits Recall subtest	85.3	14.8	64–125	100.1	16.8	68–144	.182∗∗	.077∗
*Phonological loop*								
Digit Recall subtest	96.8	17.6	56–145	115.8	18.6	85–145	.220∗∗	.179∗∗
Word List Matching subtest	101.4	14.3	78–145	111.7	17.7	80–145	.095∗	.031
Word List Recall subtest	87.2	12.1	56–113	101.9	13.3	78–131	.255∗	.188∗∗
Non-word List Recall subtest	86.7	14.3	55–117	103.5	14.3	66–128	.261∗∗	.150∗∗
*Visuo-spatial sketchpad*								
Mazes Memory subtest	79.8	14.9	56–113	81.3	15.8	56–113	.002	
Block Recall subtest	84.0	13.6	58–117	89.5	15.7	59–129	.034	

Note: ∗*p* < .05, ∗∗*p* < .001. All subtests standardised to a mean of 100 and SD of 15.

**Table 5:** Declarative memory: CMS summary scores and comparisons.

Variables	SLI			TD			Effect sizes (*partial η*^2^)			
*M*	SD	Range	*M*	SD	Range	No covariates	Covariate: working memory	Covariate: language factor	Covariates: working memory & language factor
*Declarative memory (verbal information)*										
Learning										
Word pairs	6.7	2.5	1–13	9.4	2.9	3–18	.208∗∗	.048	.070	.014
Short recall										
Word pairs	8.5	2.6	3–14	10.4	3.0	4–15	.103∗∗	.017	.010	<.001
Stories	5.7	2.5	1–12	9.1	3.1	2–16	.270∗∗	.140∗∗	.078	.049
Delayed recall										
Word pairs	7.1	2.8	1–14	9.2	2.7	3–16	.131∗∗	.038	.032	.010
Stories	6.0	2.7	1–12	9.6	3.4	2–17	.255∗∗	.093∗	.072	.024
Delayed recognition										
Word pairs	6.6	3.9	2–12	9.6	3.2	2–12	.154∗∗	.062	.042	.021
Stories	6.3	2.2	1–11	8.4	2.9	1–14	.155∗∗	.051	.037	.012
*Declarative memory (visual information)*										
Learning										
Dot locations	10.0	4.1	1–16	10.9	3.4	3–16	.014	.013		
Short recall										
Dot locations	10.0	2.8	4–14	11.2	2.4	5–14	.047∗	.053		
Short recognition										
Faces	9.1	3.1	0–17	9.0	2.3	2–15	.001	.007		
Delayed recall										
Dot locations	8.9	3.4	1–14	9.7	3.2	3–14	.017	.014		
Delayed recognition										
Faces	9.3	3.1	2–17	8.9	2.4	4–16	.004	.001		

Note: ∗*p* < .05, ∗∗*p* < .001. All subtests standardised to a mean of 10 and SD of 3.

**Table 6:** Correlations (Pearson’s *r*) between language and memory measures.

Group/language measure	Working memory			Declarative memory		Procedural memory
Central executive	Phonological loop	Visuo-spatial sketchpad	Verbal information	Visual information
*TD*						
Lexical abilities	.092	.123	−.029	.480∗∗	.251	.233
Grammatical abilities	.096	.028	.080	.235	−.096	.305∗
*SLI*						
Lexical abilities	.101	−.041	.028	.394∗	.216	−.008
Grammatical abilities	.189	.131	−.049	.305∗	.018	.112

Note: ∗*p* < .05, ∗∗*p* < .001.
